# Simulating and Optimising Quantum Thermometry Using Single Photons

**DOI:** 10.1038/srep38822

**Published:** 2016-12-15

**Authors:** W. K. Tham, H. Ferretti, A. V. Sadashivan, A. M. Steinberg

**Affiliations:** 1 Centre for Quantum Information & Quantum Control and Institute for Optical Sciences, Department of Physics, University of Toronto, 60 St. George St, Toronto, Ontario, M5S 1A7, Canada; 2 Canadian Institute For Advanced Research, 180 Dundas St. W., Toronto, Ontario, M5G 1Z8, Canada

## Abstract

A classical thermometer typically works by exchanging energy with the system being measured until it comes to equilibrium, at which point the readout is related to the final energy state of the thermometer. A recent paper noted that with a *quantum* thermometer consisting of a single spin/qubit, temperature discrimination is better achieved at finite times rather than once equilibration is essentially complete. Furthermore, preparing a qubit thermometer in a state with quantum coherence instead of an incoherent one improves its sensitivity to temperature differences. Implementing a recent proposal for efficiently emulating an arbitrary quantum channel, we use the quantum polarisation state of individual photons as models of “single-qubit thermometers” which evolve for a certain time in contact with a thermal bath. We investigate the optimal thermometer states for temperature discrimination, and the optimal interaction times, confirming that there is a broad regime where quantum coherence provides a significant improvement. We also discuss the more practical question of thermometers composed of a finite number of spins/qubits (greater than one), and characterize the performance of an adaptive protocol for making optimal use of all the qubits.

A multitude of measurement and metrology tasks have been shown to benefit, sometimes dramatically, from the substitution of certain classical resources with their quantum counterpart^1^,^2^. Examples of advances in quantum metrology range from a many-fold increase in sensitivity to phase in interferometry or polarization in polarimetry through the use of non-classical light^3^,^4^,^5^,^6^,^7^,^8^,^9^,^10^ to highly sensitive magnetometry^11^,^12^,^13^. Much in keeping with the spirit of the field, a recent analysis^14^ has considered that most primitive of metrological tasks - thermometry, or simply telling cold from hot - and found that a coherent measurement scheme can enhance thermometry beyond the traditional approach. In particular, because different-temperature baths lead not only to different equilibrium states but also to different equilibration *rates*, this means that temperature discrimination can be better achieved by comparing rates at finite times rather than asymptotic states when thermalisation is complete. Furthermore, the theory work also noted that the difference between relaxation rates for populations and coherences means that for intermediate time regimes (before full equilibration but after some characteristic time that depends on the temperatures of the baths), optimal discrimination is achieved not by probing energy only but by using quantum coherence as well.

Our measurement device is a qubit or a quantum system consisting of two levels typically called the ground (denoted 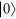) and excited (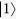) states. Furthermore, since a single spin cannot provide more than one bit of information^15^, we follow^14^ in considering the simpler task of distinguishing between just *two* (instead of a continuum of) thermal baths at two different temperatures. And whereas a traditional thermometry approach prescribes letting the thermometer fully thermalise to the given bath by waiting for a long time, whereupon some physical quantity that bears a known correlation with the temperature is measured, we allow our qubit to interact for only a finite duration before it is subjected to some measurement. The qubit interacts with the heatbath by absorbing or emitting photons from/into it. In standard treatments of spontaneous emission^16^,^17^ this photon exchange process is often described with parameters *τ*_1_, the decay time for the excited state population, and *τ*_2_, the decay time for coherences between 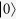 and 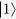. It is known that 2*τ*_1_ ≥ *τ*_2_. When there are no additional dephasing mechanisms, the equality holds so that the coherence damps away exactly half as quickly as the excited state population.

In the case of thermalisation via energy exchange with a bosonic bath, as treated in ref. 14, the collision rate grows with occupation number, and hence with temperature. *τ*_1_ and *τ*_2_ are therefore shorter for higher-temperature baths. This difference means that it is generally advantageous to wait for a finite interaction time (on the order of the thermalisation times) in order to better distinguish the two processes, rather than allowing the qubit to fully thermalise with the bath. In particular, for high temperatures, the asymptotic populations are very similar, while the thermalisation rates may be quite different. Since coherences decay twice as slowly as populations, the time of optimal distinguishability occurs roughly twice as late for initially coherent states (which decay principally at *τ*_2_) as for initially incoherent states (which decay at *τ*_1_). As a consequence, one can show that beyond a certain critical time, an initial state with some coherence always makes for a more sensitive thermometer. Similar results are expected to hold in any case where thermalisation occurs faster with higher-temperature baths (for instance, via collisions with Maxwell-Boltzmann distributed gas molecules), but not if the thermalisation rate were fixed and temperature-independent, as in commonly used phenomenological models of thermal conductance. (For the interested reader, we give a mathematical argument in the Supplementary, as to why these observations are true). This work therefore aims to experimentally demonstrate this metrological advantage, along with an extension to the more practically relevant case where one is not restricted to the use of a single qubit.

In the Bloch sphere representation, every single qubit state corresponds uniquely to a 3-vector on or within the unit sphere. Conventionally, the excited state is represented as +*Z* and the ground state as −*Z*. Maximally coherent states lie on the *xy*-plane, usually with 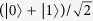 at +*X*. The thermalisation of the qubit in this picture can be thought of as a trajectory from its initial Bloch vector (usually a unit vector on the surface of the unit sphere if the initially prepared state is pure) to its final point on the *z*-axis. To aid the reader in visualising this, Fig. 1 shows trajectories of a qubit initialised in +*Z*, −*Z*, and +*X*, interacting with a bath of temperature 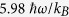 or 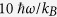. For the ±*Z* initial states, the evolution of the qubit is strictly along the *z*-axis, so only the *z* component (denoted *s*_*z*_) is shown. When the qubit is initialised to +*X*, however, both the coherence (the *x*-component, labeled *s*_*x*_) and the excited state population (the *z*-component) relax with time so both are shown in Fig. 1(c) over a range of discrete times.

The yardstick by which we will characterise the performance of our qubit thermometer is the probability with which we mis-identify our bath, either by mistaking a hot bath for a cold one or vice versa. Since a thermalised qubit is almost always in a mixed state (a statistical mixture of pure states), this error probability or *p*_*e*_ never vanishes. For example, a fully thermalized qubit at temperature *T* = ∞ is in a state that is an equal statistical mixture between 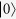 and 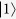 whereas at *T* = 0 it is in the ground or 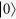 state. Suppose we now identify our heatbath as the hot one if and only if a measurement on the qubit finds it in the excited state. Although we will never misidentify the *T* = 0 bath, the *T* = ∞ bath yields the excited state with 50% probability so we stand to misidentify it half the time! Assuming that a given bath is chosen from *T* = ∞ and *T* = 0 with equal likelihood, our overall error probability, *p*_*e*_, is 1/4. Tasks such as the one just described are aptly called state discrimination. Conveniently, *p*_*e*_ in state discrimination is well-known to be related to 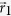 and 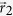, Bloch vectors corresponding to the states being discriminated, as follows^18^,^19^:


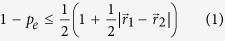


where the norm is to be understood as the usual Euclidean/Cartesian distance between vectors 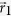 and 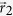. Returning to our example with *T*_*hot*_ = ∞ and *T*_*cold*_ = 0, the completely mixed state corresponds to 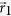 = 〈0, 0, 0〉 and the ground state to 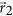 = 〈0, 0, −1〉, so |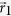 − 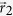
| = 1 implying once more that *p*_*e*_ ≥ 1/4. It is important to stress that while a larger Euclidean distance between Bloch vectors implies that a lower error probability is achievable in principle, actually saturating the inequality to achieve the lowest possible error requires that we select the correct basis during measurement. In our example above, measuring if the qubit is in the state 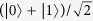 would have yielded a “yes” answer with probability 50% for *both T* = 0 and *T* = ∞, giving us no information at all about the bath!

## Experimental Design

### Emulating thermalisation with photons

Since we have opted to emulate the effects of thermalisation on a photonic polarisation qubit, we must first understand how thermalisation affects qubits in general. In the absence of extraneous damping processes (e.g. mechanical collisions), a qubit that interacts with a thermal reservoir by photon exchange alone can be treated as a system that emits or absorbs a photon into/from the reservoir with some probability. Such a process is well-modeled by a generalised amplitude damping (GAD) channel, which is defined in the standard operator-sum representation as follows^19^:


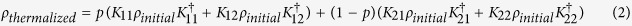



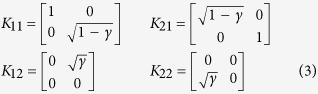


Equivalently we can write the above process in terms of its action on the Bloch vector:


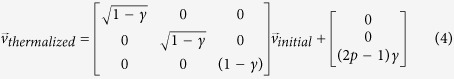


In words, the Kraus operators describe two physical sub-processes: *K*_11_ and *K*_12_ jointly describe a sub-process in which the qubit in 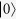 *absorbs* a photon from the reservoir with probability *γ* thereby transitioning to 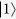. *K*_21_ and *K*_22_ describe the opposite sub-process in which a photon is *emitted* into the reservoir again with probability *γ*. A thermalising qubit is merely one which undergoes the first sub-process (absorption) with probability *p* and the second (emission) with probability 1−*p*. The probability *p* in turn is determined by the bath temperature. To see this, suppose our qubit states 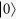 and 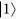 have energies *E*_0_ and *E*_1_ respectively. After fully thermalising, we expect our qubit, which is now in a mixture of 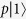 and (1−*p*)|0〉, to obey thermal statistics. We expect:


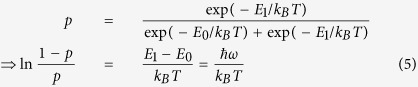


so *p* → 0 as *T* → 0 whereas *p* → 1/2 as *T* → ∞. It is also well-known that for a bosonic thermal reservoir, the Planck distribution implies an average occupation number 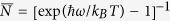. Thus, in terms of 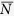, we can write *p* more compactly as 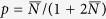.

The damping parameter *γ* is related to the interaction time with the bath, *t*, and temperature, *T*, as follows: *γ* = 1−exp(−*t*/*ξτ*_*sp*_), where *τ*_*sp*_ is a timescale characteristic of the coupling between qubit and bath. Here,


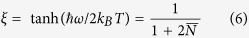


is a unitless quantity that encodes the bath temperature. Note from equation 6 that *ξ*, and therefore the relaxation rate of the qubit’s excited state population and coherence, is temperature dependent. This, coupled with the fact that the difference of two exponential functions with different exponents is *not* monotonic, means that the Euclidean distance between resultant states for the hot vs cold baths is larger (and our thermometer more sensitive) when the interaction time *t* is finite (partially thermalised) as opposed to infinite (fully thermalised).

Since in our case we are merely emulating thermalisation, *ω* which defines the mode through which the qubit is coupled to the bath, is ill-defined. We shall therefore specify temperature in terms of 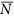 and *ħω*/*k*_*B*_. Likewise, we do not have an intrinsic timescale by which to specify *τ*_*sp*_. All times *t* will be specified in units of *τ*_*sp*_. As we’ll see, encoding the qubit in two orthogonal polarisations of a photon (call them 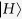 and 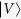) makes it possible to implement this compactly in a tabletop experiment. In keeping with the cases considered in Jevtic *et al*.^14^, unless otherwise mentioned, we will henceforth consider two baths with temperatures 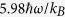 and 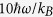.

### Implementation

**Figure 2:**
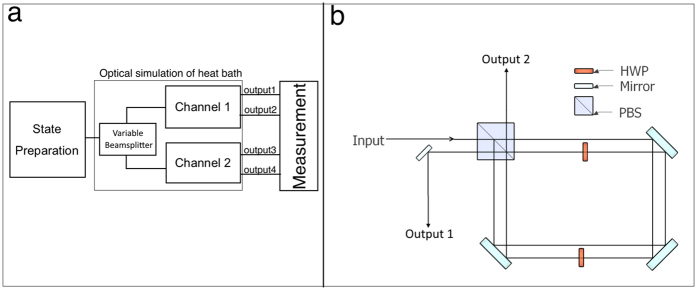
Experimental Scheme. (**a**) Block schematic of the experimental setup for channel emulation. (**b**) Drawing of an optical implementation of the “channels” block. Two similarly constructed copies of the channel fed a via splitter that stochastically switches between them implements a simulation of a thermal bath. The “switching” is done via a variable beamsplitter (VBS), whose coefficient of reflection/transmission can be modulated.

Our experimental setup begins with our light source, a continuously pumped type-II spontaneous parametric down-conversion (SPDC) crystal. One photon of the SPDC pair is sent directly to a single-photon counting module (SPCM) to act as herald whereas the other is sent through a polariser, followed by a quarter-wave plate (QWP) and then a half-wave plate (HWP). Together, these form the state preparation block of inset (a) Fig. 2, which allows us to encode our qubit onto the polarisation of the 810 *nm* SPDC photons.

To simulate thermalization, we make use of an optical circuit having the capacity to simulate any valid single-qubit quantum channel (precisely, completely positive trace-preserving or CPTP maps). The design of this circuit was inspired by Sanders *et al*.’s theoretical results^20^, which were in turn based on mathematical work by Ruskai *et al*.^21^. A similar optical circuit has already been shown to accurately simulate a wide variety of single-qubit quantum channels^22^. Our design consists of a variable beamsplitter (VBS), followed by two subsequent interferometers (labeled channels 1 and 2 respectively in Fig. 2). The optical design of each channel is shown in inset (b) of Fig. 2. Light that is incident on each channel is split at a polarising beamsplitter (PBS) so that each of two orthogonal polarisations travels along spatially separated counter-propagating paths. Each path contains a HWP, allowing the polarisations to be rotated independently before being recombined at the PBS. It is easy to see how this intra-interferometer rotation allows us to emulate a damping channel. Suppose we leave the 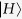 polarisation within the interferometer unrotated but leave the HWP within the |*V*〉 path at 45° so that 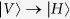. Such a setting guarantees that the output of the channel is *always* 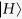 regardless of input polarisation - hence a full damping channel with *γ* = 1. Less extreme settings realise the full range of damping channels. More formally, it is easy to show that each channel is well described by 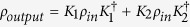 with:


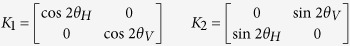


Here *θ*_*H*_ and *θ*_*V*_ are angles of the fast axis of the half-wave plates in channels 1 and 2, acting on the horizontal and vertical paths respectively. Setting *θ*_*H*_ = 0 and sin 2*θ*_*V*_ = 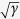 implements *K*_11_ and *K*_12_ in equation 3, whereas setting *θ*_*V*_ = 0 and 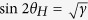 implements *K*_21_ and *K*_22_.

Although the inner workings of the VBS are not shown, it is identical in design to the channels except for the fact that there is a single HWP (instead of two independent ones) acting on both counter-propagating paths. This restricts the action of the VBS to a *fixed* unitary that can be subsequently undone for all input states with a simple HWP placed outside the VBS. Notice that if we now set the HWP in the VBS to cos^2^ 2*θ*_*VBS*_ = *p*, both channels work in tandem to fully implement the map in equation 2. Thus, we have a fully tunable means of emulating thermalisation with a polarisation qubit.

Finally, the measurement block consists of a QWP →  HWP →  PBS sequence. The four output states (two from each channel, one from each Kraus operator) are mixed and sent through this measurement sequence and then onto an APD and coincidence counter. In practice, losses and imperfections in optical components means that mixing the four output states *before* the measurement sequence is impractical since it precludes the possibility of compensating with post-processing. Instead, we opted to send each one to two APDs after the measurement sequence (they are further split at the PBS into 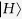 and 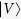) and then only tracing over them in post-processing.

### Experimental Data

**Figure 3:**
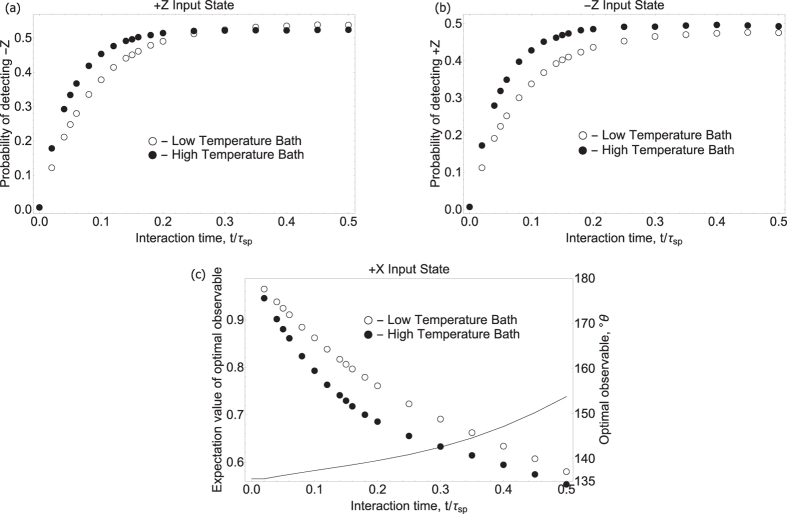
Plots of inferred detection probabilities. For each input state and channel setting, we computed and performed the optimal measurement for discrimination between the final states from each heat bath. **(a)** Detection probability of the −*Z* observable (i.e. 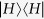) when input state is +*Z* (or 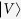). **(b)** Detection probability of +*Z* observable (i.e. 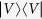) when input state is −*Z* (or 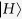). **(c)** Detection probability for some optimal measurement given input +*X* (or 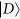). Although for ±*Z* input states the optimal observable is fixed, in the +*X* case it varies with interaction time. In this plot, the optimal observable is parametrised as: 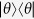 where |*θ*〉 = cos *θ*|*H*〉 + sin *θ*|*V*〉. For reference, the relaxation times for the hot bath are *τ*_2_ = 2*τ*_1_  = 0.05*τ*_*sp*_. For the cold bath, they are *τ*_2_ = 2*τ*_1_ =  0.083*τ*_*sp*_.

To ensure that our channels are emulating the desired thermalisation process, we characterise it via full process tomography^23^,^24^,^25^,^26^. Throughout stretches of data-taking, we re-characterise periodically (approximately every 30 minutes, the minimum time in which visibility of interferometers are likely to have dropped appreciably) and realign optics as necessary. We proceed to prepare states −*Z* (or 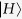), +*Z* (or 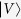), and +*X* (or 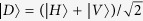). We emulate the heat baths specified in ref. 14, with 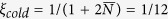 and *ξ*_*hot*_ = 1/20. These correspond to temperatures of 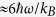 and 10*ħω*/*k*_*B*_ respectively. We further set the channels to emulate interaction times ranging from *t* = 0*τ*_*sp*_ to *t* = 0.4*τ*_*sp*_, where asymptotics have yet to dominate and dynamics are non-trivial. The time steps are sampled unevenly because: a) at large *t*’s, we were limited by the precision of our motorised rotation stage (~0.2) whereas b) at small *t*’s, we restricted ourselves to Δ*t* ≥ 0.2*τ*_*sp*_ in order to maintain a reasonable sampling stepsize. For each channel setting, we chose to measure along a basis prescribed by static state discrimination strategies to be optimal (i.e. select a projector *M* s.t. equation 8 is minimized, discussed in next section). We counted photons for 10 seconds per measurement, which yielded measurement “shots” that consist of ~40,000 photons apiece. Although the thermometry scheme discussed above is intended for single qubits, we have opted to use a bright source and long count durations in order to infer Tr(*ρM*), the probability of a successful outcome of the projector, *M*, in our chosen measurement basis. Since we expect the number of coincidences to be binomially distributed, this inferred detection probability tends to the true single-photon probability with diminishing uncertainty as the total photon number becomes large. Figure 3 show this inferred probability. Note that each point in the plots represents an average over many sets of data taken under identical experimental conditions (9 sets for the +*Z* case, 10 sets for +*X*, and 4 for −*Z*, where a hardware issue forced us to discard 5 sets of data; discarded sets are reported in the Supplementary, for completeness).

**Figure 4:**
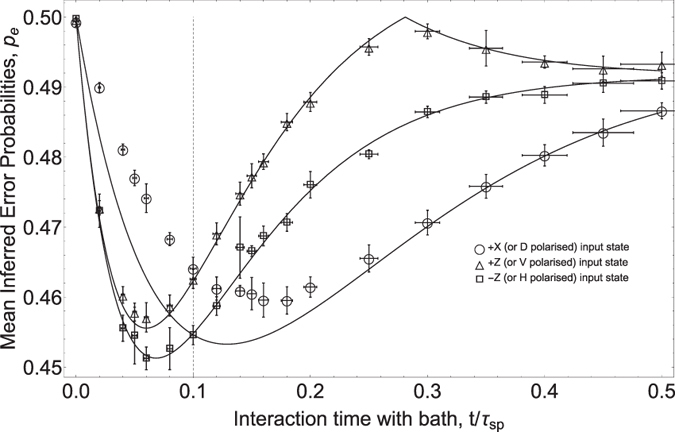
Inferred Error Probabilities in discriminating between final states from two heat baths. Solid lines are theory curves. Points are experimentally inferred error probabilities. Vertical error bars indicate standard deviation or spread range over multiple repetitions for a given channel setting and input state. Horizontal error bars indicate uncertainty in channel setting due to finite precision of motorised rotational mounts. Relaxation times for the hot bath are *τ*_2_ = 2*τ*_1_  = 0.05*τ*_*sp*_. For the cold bath, they are *τ*_2_ = 2*τ*_1_ = 0.083*τ*_*sp*_. The vertical dashed line is a guide to the eye to indicate where theory predicts a change in the optimal initial state changes, in this case from −Z to +X.

In order to compare with theory, we must deduce an effective discrimination error probability *p*_*e*_ from the above detection probabilities. Now given two states *ρ*_1_ and *ρ*_2_ (in our case these are the state of the qubit after interacting for some time, *t*, with the hot/cold bath respectively), *p*_*e*_ can be computed as:


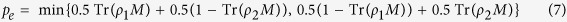


where 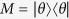, 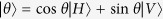, and Tr(*ρM*) are detection probabilities shown in Fig. 3. The results are shown in Fig. 4. Overlayed are theory curves deduced (via equation 1) from the Euclidean distances between final states of the ideal GAD. Reiterating theoretical results mentioned above, we see that after approximately two thermalisation times (*t* ~ 0.1*τ*_*sp*_) the coherent state +*X* outperforms the incoherent ones ±*Z*. Note that although −*Z* appears to be *globally* optimal (i.e. has a lower *p*_*e*_ at *t* ~ 0.07*τ*_*sp*_ than other states at any time), this is peculiar to our choice of temperatures and is not always the case. A specific example in which coherence is necessary for achieving the global optimum is given in the Supplementary Information.

While the behaviour of *p*_*e*_ shows good agreement with theory for ±*Z* input states, the +*X* case shows discrepencies in the region *t* ≤ 0.2*τ*_*sp*_. This can be ascribed to the fact that our interferometers have finite visibility. This has the effect of mapping some amount of coherence between |*H*〉 and 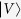 to an incoherent mixture and is completely analogous to extraneous dephasing processes (e.g. atomic/molecular collisions etc.) that we did not consider in our thermalisation model. Note that ±*Z* input states do *not* experience interference effects as they traverse the channels - these states end up traveling through the interferometer via just *one* of the two possible counter-propagating paths and have nothing to interfere with when they re-emerge at the PBS. The ±*Z* states are therefore not susceptible to imperfect interferometer visibility. The same cannot be said of the +*X* state. The interferometers that comprise our channels have typical visibilities ≥95%, though due to the lack of active stabilization, realignment can become necessary from time to time.

**Figure 5:**
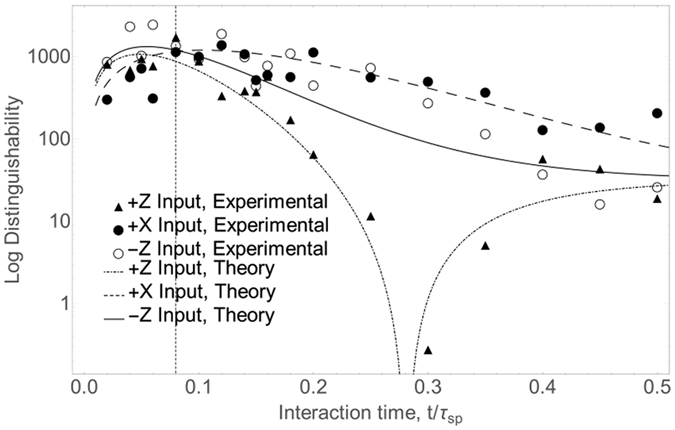
Plot of distinguishability in the outcome of our optimal observable (see Fig. 2). Higher points indicate better distinguishability. Means and variances were computed over multiple sets of comparable experimental data. Average photon number in all measurements was ≈40,000. The vertical dashed line is a guide to the eye to indicate where theory predicts a change in the optimal initial state, in this case from −Z to +X.

**Figure 6:**
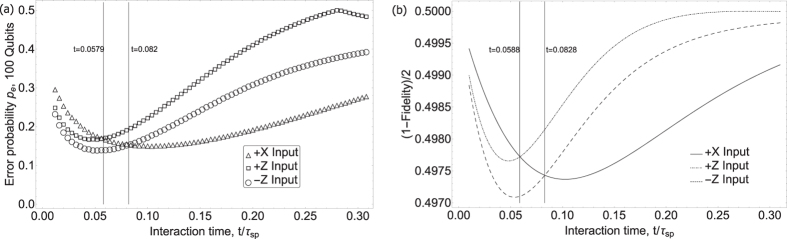
Performance measures for a multi-qubit thermometer. (**a**) Numerically computed error probabilities for 100 qubits. **(b)** The fidelity between states being discriminated (identical states have fidelity 1 whereas orthogonal states have fidelity 0). The vertical lines are guides to the eye to indicate where one initial state surpasses another in discrimination optimality (the left vertical line marks the time when +X state becomes preferable to +Z, whereas the right vertical line indicates when +X becomes preferable to −Z).

While the notion of the Euclidean distance and the error probability are the correct figures of merit to use for state discrimination tasks, the former applies strictly to single-qubit states whereas the latter becomes increasingly difficult to compute for large numbers of qubits. An alternate measure is the distinguishability, often used as a measure of the ease with which two distributions can be distinguished. It is defined as the squared difference of the means of the two distributions, divided by their variance:


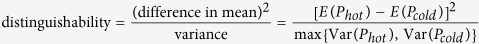


where *P*_*hot*_ and *P*_*cold*_ are the binomially distributed outcomes of some projector observable given the output state from each of the heat baths (i.e. the probabilities shown in Fig. 3). A plot of this measure is shown in Fig. 5. We attribute the noise in the experimental points in this figure to the fact that relatively few sets of data (9 sets for +Z and +X inputs and 4 sets for −Z) were used to infer the variances. Although qualitatively quite similar to the *p*_*e*_ plot in Fig. 4 (i.e. the +*X* state remains optimal after some time, while −*Z* is optimal if time is not a constraint), the two measures disagree for example on the cutoff times at which the optimal input state changes. A clear advantage for +*X* is seen at *t* > 0.08*τ*_*sp*_ (from theory curves in Fig. 5), whereas in the Euclidean distance or single-qubit *p*_*e*_ case it is seen after *t* > 0.1*τ*_*sp*_. This discrepancy leads one to suspect that the optimal input state for our thermometer in a multi-qubit scenario is different from the single-qubit case. In order to obtain a more rigorous measure of many-qubit distinguishability, we numerically computed the error probability *p*_*e*_ for 100 qubits. This is shown in Fig. 6 along with the fidelity, 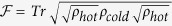 where *ρ*_*hot*_ and *ρ*_*cold*_ are final states from the hot and cold baths respectively. The quantity 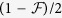 has been shown^18^ to bound *p*_*e*_ from above in the limit of an asymptotically large number of qubits. Again, the differences between a single qubit and many qubits is clear - the crossover between −*Z* and +*X* in Fig. 6(b) occurs at *t* = 0.0828*τ*_*sp*_. In the next section, we treat the many qubit case more carefully.

### Multi-qubit extension and adaptive state discrimination

#### A Bayesian approach

While a single-qubit thermometer is conceptually interesting, it is obviously of more practical relevance to consider a thermometer composed of many spins, but potentially a limited, fixed number. Already, the static strategy - doing the same thing on all *N* copies of a qubit - yields better binomial statistics the larger *N* is. Let’s return to the example in the Introduction, of distinguishing *T* = 0 from *T* = ∞. The best static strategy for *N* qubits in that case is to measure along some optimal axis for all qubits and conclude that the bath is at *T* = 0 if and only if *all* measurement outcomes are 0. In that case, 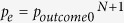.

One can often do better^27^,^28^ by allowing for an adaptive strategy (say by changing the measurement basis for each qubit), a possibility that was investigated by Wiseman *et al*.^29^. To facilitate further discussion, we now restate the discrimination problem in slightly more formal terms. Whereas in the preceeding discussion we assumed that the bath was equally likely to be in *T*_*hot*_ or *T*_*cold*_, we now allow each to occur with different prior probabilities (call them *π*_1_ and *π*_2_ where *π*_1_ + *π*_2_ = 1). We also assume that for a given interaction time *t*, the output states from baths *T*_*hot*_ and *T*_*cold*_ are known to be *ρ*_1_ and *ρ*_2_ respectively. The problem is to find an optimal *strategy* that yields *p*_*e, min*_ = min_*strat*_ {*p*_*e, strat*_}, the lowest error over all possible strategies. A *strategy*, in turn, is a combination of a measurement (observable *M*) and a *threshold* (*k* ∈ *Z*) beyond which one concludes that the output state is *ρ*_1_ (or *ρ*_2_). In this language, the error probability for each case (assuming we employ a strategy non-adaptively, i.e. the same *M* for all *N* qubits):


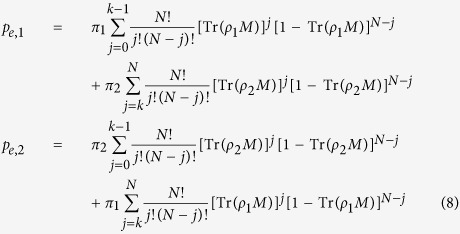


The optimal error probability is therefore computed as *p*_*e*,*min*_ = min_*k*,*M*_{*p*_*e*, 1_, *p*_*e*, 2_}. If *ρ*_1_ and *ρ*_2_ are two single-qubit states, the minimization over *M* reduces to a minimization over one real parameter (i.e. the measurement angle *θ*; we can assume *ρ*_1_ and *ρ*_2_ both lie on the real plane of the Bloch sphere - else we rotate them onto it).

In the adaptive case where *θ* may vary with each copy of *ρ*, the above no longer holds. Instead, consider the following approach: suppose on the first measurement *M*_1_, we obtain a successful (√) outcome. We update our state of knowledge as follows:


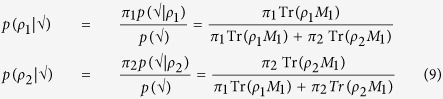


We then take these as the “updated” priors: 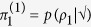 and 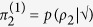 where the superscript indicates that they are post-measurement-1. Had the outcome of *M*_1_ been negative, we would simply have replaced every instance of Tr(*ρM*) in equation 9 with 1 − Tr(*ρM*). We then return to equation 8 to minimize *θ* substituting 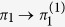 and 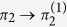. Now armed with *M*_2_, we proceed to repeat the procedure on the next copy of *ρ*. After all qubits are measured, we finally choose the state associated with the larger of 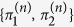 as the output of our discrimination procedure. In ref. 29, it was shown that such a Bayesian prior update strategy is not only better than the static one but is in fact optimal if *ρ*_1_ and *ρ*_2_ are pure states! And although it isn’t the optimal strategy for an arbitrary mixed state, it nevertheless performs better than the static approach.

#### Testing Adaptivity

To test the adaptive approach, we set the channel to identity (it simply preserves all input states; in practice this meant setting *p* = 0 in the VBS and *θ*_*H*_ = *θ*_*V*_ = 0° for the channels). The states to discriminate were 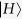 and 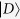. These states were chosen (instead of outputs of the thermalisation channel described in preceeding sections) because the identity channel much easier to control, allowing us to avoid potential confounding factors when demonstrating the benefits of adaptivity. The identity channel requires only one of channels 1 and 2 to be active and so does *not* require LCWP calibration or careful alignment of the VBS, among other things.

**Figure 7:**
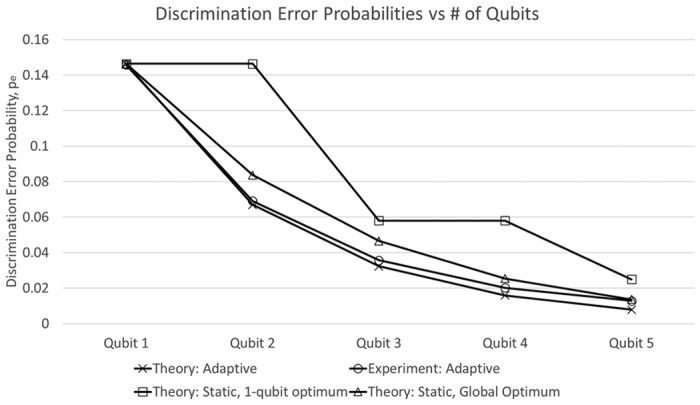
Plot of error probability in discriminating 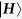 from 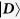, by number of qubits for various strategies.

As before, we began by validating the channel via process tomography and then proceeded to measure along bases specified by equations 8 and 9 above. In an actual adaptive scenario, one ideally updates one’s measurement setting as each photon is detected. However, our motorised waveplate mounts are relatively slow making it difficult for us to adapt our measurement setting conditioned on individual detection events. Instead, we have chosen to measure along *all* bases that are prescribed by our Bayesian update strategy given *all* possible detection outcomes. This allowed us to map out the full tree of possible outcomes along with the probability of occurrence for each node. Such a map of outcome probabilities allows us to confirm that the Bayesian update rule is valid, even if we can’t directly emulate adaptivity. Figure 7 shows a plot of how the error probability, *p*_*e*_, scales with number of qubits in the various scenarios.

Figure 7 shows the resulting error probabilities, *p*_*e*_, for various strategies. Theoretical predictions and experimentally derived values for our strategy, detailed above, are plotted in blue and red, and are labelled “Adaptive”. For comparison, theory predictions for two static (non-adaptive) cases are shown. The “1-qubit optimum” naively uses a measurement basis that is optimal for just 1 qubit, and repeats it as necessary. On the other hand, in the “Global Optimum” approach, one is assumed to have been told the total number of qubits available, and a *static* measurement angle that is optimal for the given number of qubits is computed and used.

Evidently, the *adaptive* multi-qubit scenario offers the benefit of lower error probabilities. The absolute reduction is particularly pronounced for the first few additional qubits, when *p*_*e*_ is still relatively large. The deviation between our data and theoretical prediction for *p*_*e*_ becomes pronounced as the number of qubits *N* becomes large. The probability tree that we must reconstruct grows quickly with *N* (generally, 2^*N*^ branches) and so does the precision with which we must set our measurement basis in order to maintain an advantage over the non-adaptive approach. In our case, we are limited by our motorised waveplate mounts to an angular precision no better than ±0.2°.

Also of note, our adaptive strategy makes no assuption about the total number of qubits and continues to work in a “rolling” fashion even if, midway through the scheme, we were suddenly told that more qubits have suddenly become available. This is in stark contrast to the “global optimum” static strategy, where a favorable scaling is only possible given full knowledge of just how many qubits there are.

## Conclusions

In summary, we have simulated the thermal equilibration of a spin by using a construction of a universal emulator for quantum channels. This has allowed us to confirm Jevtic *et al*.’s^14^ theoretical conclusions that for thermalisation with a bosonic bath, optimal temperature discrimination occurs at early times rather than in the asymptotic limit, and that for most interaction times, a thermometer initialized in a coherent superposition state outperforms one prepared in the ground state. In our case, this advantage translates to a maximum reduction of the error probability, *p*_*e*_, from 47.99% (theory, or 48.04% experimental) to 46.19% (46.55% experimental) when discriminating between temperatures 5.98*ħω*/*k*_*B*_ and 10*ħω*/*k*_*B*_ after letting the qubit interact with the bath for *t* ≈ 0.23*τ*_*sp*_. This is a 90% (76% experimental) increase in the improvement over a purely random guess (*p*_*e*_ = 50%). Furthermore, after just *t* ≈ 0.068*τ*_*sp*_, the error probability is reduced, relative to a fully thermalised qubit, from 49.17% to 45.13% or approximately a 5.8 fold advantage in the improvement over random guess. We discuss the origin and limitations of this behaviour, and study the extension to the case of a thermometer composed of a finite number of spins, showing the advantages of an adaptive measurement strategy. We note that there are important differences between the optimization problem for single and multiple spins, but conclude that quantum coherence retains an advantage even in the latter case. This is a new example of a quantum metrological advantage, and may prove important for making accurate measurements of thermal properties of quantum systems with limited resources or limited disturbance.

*Note*: During preparation of this manuscript we became aware that similar work was being pursued by Mancino *et al*.^30^.

## Methods

The pump laser for our SPDC source is a 120 mW continuous wave (CW) 405 nm laser diode produced by Mitsubishi (Thorlabs Part ML320G2-11). Driven by this laser is the SPDC crystal itself, a 2 mm thick beta-Barium Borate (BBO) crystal phase-matched with an opening angle of 3°. Light from the SPDC crystal is filtered with 10 nm bandpass filters (sourced from ASAHI). The resulting typical total coincidence rate (taking into account all losses in the experiment) is ≈4000 pairs per second. Our single-photon detectors are avalanche photodiodes (APDs) manufactured by Perkin Elmer (now Excelitas Technologies). We used a combination of a 4-detector module (SPCM-AQ4C) and 4 single-detector modules (SPCM-AQR-13). Both have similar performance parameters (photon detection efficiency ~50% at 800 nm and dark count rate 500/s). TTL signals from the APDs are counted by a homebuilt coincidence counting circuit (details can be found at: http://bit.ly/2eGo1L811-channel coincidence counter project page). Half- and quarter-waveplates (HWP and QWP) in the experiment were sourced primarily from Foctek Photonics, liquid-crystal waveplates from BolderVision Optik, and miscellaneous optics (mirrors, polarising beamsplitters (PBS), etc) from Thorlabs.

During the course of this experiment, we came to realise that the process fidelity of our channels were crucially dependent on the purity of the polarisation extinction of the PBS used in the construction of each channel (as shown in Fig. 2). Typical PBS extinctions were approximately 1000:1 and 100:1 for the transmitted and reflected ports respectively. We compensated for the poorer polarisation purity of reflections at the PBS by placing an additional polariser at the output port labeled “Output 1” in Fig. 2 panel (b), oriented to transmit only V-polarised light. It is easy to show that if we set the HWP in the path propagating counter-clockwise within the Sagnac interferometer to 0°, this eliminates “leaked” polarisation to 1st order while preserving the desired qubit operation. As discussed in previous sections, this is just the HWP setting we need for amplitude damping channels.

## Additional Information

**How to cite this article**: Tham, W. K. *et al*. Simulating and Optimising Quantum Thermometry Using Single Photons. *Sci. Rep.* **6**, 38822; doi: 10.1038/srep38822 (2016).

**Publisher's note:** Springer Nature remains neutral with regard to jurisdictional claims in published maps and institutional affiliations.

## Supplementary Material

Supplementary Information

## Figures and Tables

**Figure 1:**
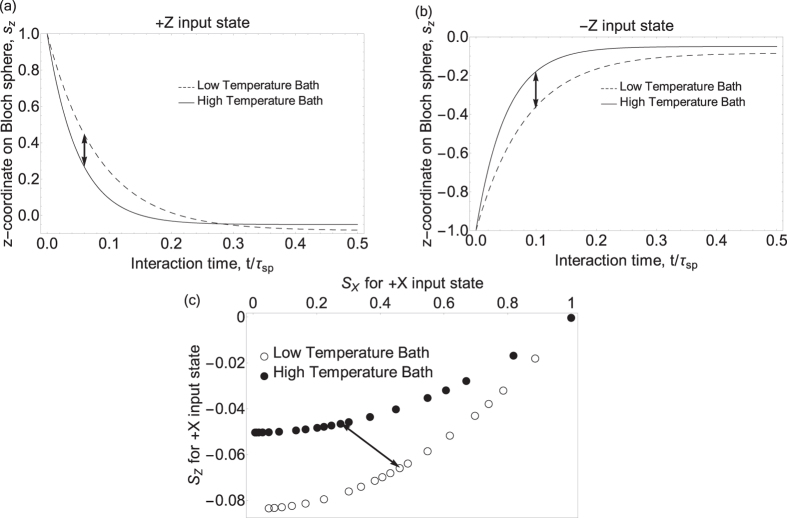
Bloch vector components vs interaction time. Theoretically computed components of the Bloch vector after thermalizing for *t* seconds, **(a)** given a +*Z* input state, **(b)** −*Z* input state, and **(c)** +*X* input state. For this latter case, both *s*_*z*_ and *s*_*x*_ are shown. The bath temperatures are 5.98*ħω*/*k*_*B*_ and 10*ħω*/*k*_*B*_. At *t* = 0 the state begins at the rightmost point of the bottom plot. Each subsequent timestep is shown as a pair of points, one each for high and low temperatures respectively. Arrows indicate where the greatest separation occurs.
