# Supplementary material for: Simulating and Optimising Quantum Thermometry Using Single Photons
Source: Sci Rep. 2016 Dec 15;6:38822. doi: 10.1038/srep38822 (PMC5156908; doi:10.1038/srep38822)
Supplement: Supplementary Information [file srep38822-s1.pdf]

Simulating and Optimising Quantum Thermometry Using Single Photons

W.K. Tham[1,*], H. Ferretti[1], A.V. Sadashivan[1], and A.M. Steinberg[1,2]

[1]Centre for Quantum Information & Quantum Control and Institute for Optical Sciences, Department of Physics, University of Toronto, 60 St. George St, Toronto, Ontario, Canada, M5S 1A7
[2]Canadian Institute For Advanced Research, 180 Dundas St. W., Toronto, Ontario, Canada, M5G 1Z8
[*]wtham@physics.utoronto.ca

Supplementary

The ground state is not always optimal

In Jevtic et al it was argued that if one is allowed to measure the qubit at any time, then the optimal state to prepare is always the ground ($-Z$) state. We wish to point out that that fact is peculiar to a certain set of temperatures for the hot/cold bath. By way of a counter-example, we show below that there exists pairs of bath temperatures for which this isn't true.

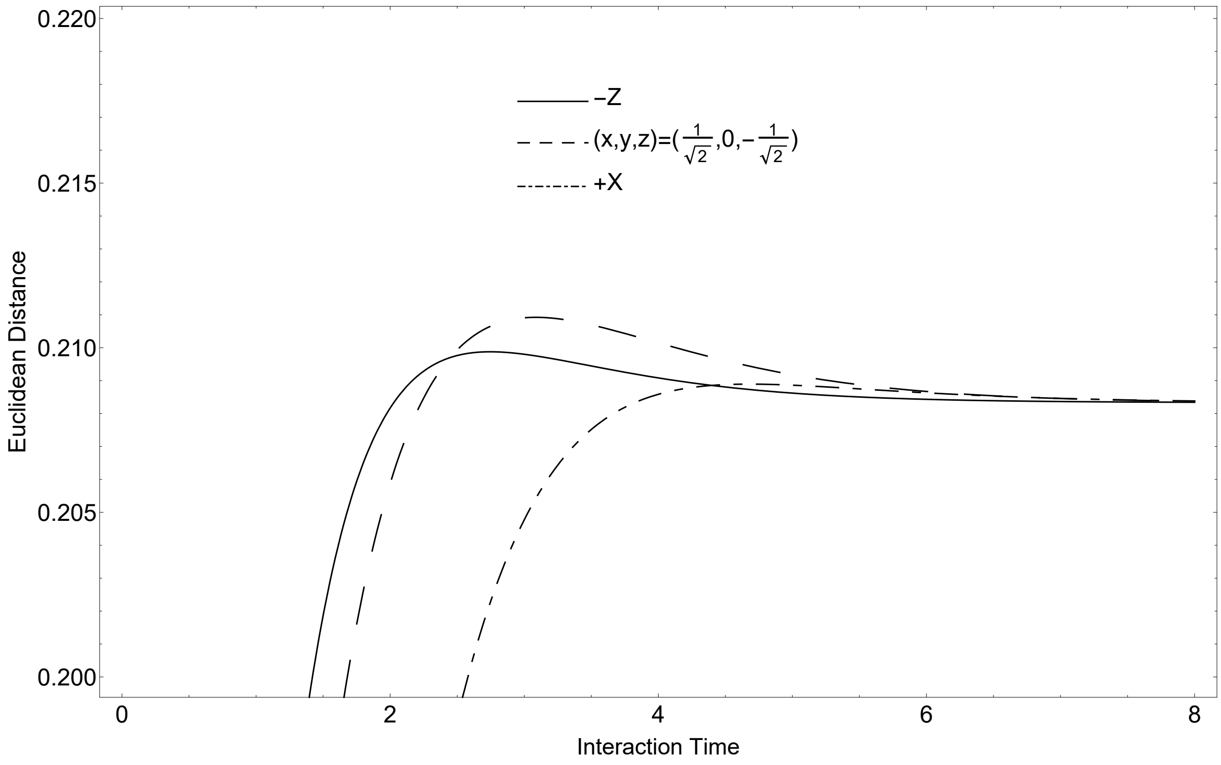

Figure 1. Plot of the Euclidean distance (larger is better) versus interaction time with bath, for three different input states.

For the above plot, we have arbitrarily chosen bath temperatures: $T_{cold} = 0.417\hbar\omega/k_B$ and $T_{hot} = 0.681\hbar\omega/k_B$ (these correspond to mean boson numbers of $\overline{N} = 0.1$ and $\overline{N} = 0.3$). Evidently, the state $(\sin(135\pi/360)|0\rangle + \cos(135\pi/360)|1\rangle)$ (i.e. Bloch vector $\vec{r} = \left\langle 1/\sqrt{2}, 0, -1/\sqrt{2}\right\rangle$) performed better at its peak than either the ground state $-Z$ or the maximally coherent state $+X$.

What state is optimal when?

As described in equations 2, 3, and 4, the decay in coherence and population are exponential in time but with the latter

occurring at twice the rate of the former. Consider the following expressions:

$$\begin{aligned}
\Delta_x &= e^{-at} - e^{-bt} \\
\Delta_z &= e^{-2at} - e^{-2bt} \\
\therefore \Delta_x - \Delta_z &= \left(e^{-at} - \left(e^{-at}\right)^2\right) - \left(e^{-bt} - \left(e^{-bt}\right)^2\right)
\end{aligned}$$

Without loss of generality, suppose $b > a$. Then at sufficiently large times, both e^{-at} and $e^{-bt} \ll 1$. In which case $\Delta_x - \Delta_z \approx e^{-at} - e^{-bt} > 0$. Now if Δ_x and Δ_z truly were Euclidean distances between states resulting from interaction with the hot and cold baths, given input states $+X$ and $-Z$ respectively, we'd now conclude that the $+X$ state performs better (because $\Delta_x > \Delta_z$). However, the final state for heatbaths of different temperatures aren't identical, so the argument needs further refinement. From equation 4, it is evident that the following modifications are necessary (we denote the corrected Euclidean distances as $\Delta_x^{'}$ and $\Delta_z^{'}$):

$$\begin{aligned}
\Delta_x^{'} &= \sqrt{\Delta_x^2 + D^2} \\
\Delta_z^{'} &= \Delta_z + D \\
\text{where } D &= \frac{1}{2a}\left(1 - e^{-2at}\right) - \frac{1}{2b}\left(1 - e^{-2bt}\right)
\end{aligned}$$

$$\begin{aligned}
\Delta_x^{'2} &= \Delta_x^2 + D^2 \\
\Delta_z^{'2} &= \Delta_z^2 + D^2 + 2\Delta_z D
\end{aligned}$$

$$\begin{aligned}
\therefore \Delta_x^{'2} - \Delta_z^{'2} &= \left(e^{-at} - e^{-bt}\right)^2 - \left(e^{-2at} - e^{-2bt}\right)^2 - 2\Delta_z D \\
&= \left(e^{-at} - e^{-bt}\right)^2 - \left(e^{-2at} - e^{-2bt}\right)^2 - \left(e^{-2at} - e^{-2bt}\right)\left[\frac{1}{a}\left(1 - e^{-2at}\right) - \frac{1}{b}\left(1 - e^{-2bt}\right)\right]
\end{aligned}$$

Now if we take the expression in the limit where $t \gg 1$ so that and $e^{-bt} \ll e^{-at} \ll 1$, then we can keep only the e^{-2at} terms. Furthermore, from equation 5 it should be clear that for any finite temperature (i.e. $\bar{N} > 0$), $0 < \xi < 1$, so that both a and $b > 1$. Thus,

$$\begin{aligned}
\Delta_x^{'2} - \Delta_z^{'2} &\approx \left(1 - \frac{1}{a} + \frac{1}{b}\right) e^{-2at} > 0 \\
\therefore \Delta_x^{'} &> \Delta_z^{'}
\end{aligned}$$

and we find that for two heatbaths at different *finite* temperatures, the $+X$ state always performs better than the $-Z$ state at sufficiently long interaction times.

We can in fact extend our treatment to states other than $+X$ and $-Z$. Again returning to equation 4, for an initial state represented by Bloch vector $\vec{v}_0 = \langle \alpha, 0, \beta \rangle$ and heatbaths characterised by $1/\xi_{cold} = a$ and $1/\xi_{hot} = a + \delta$ (where both a and δ are positive and $a > 1$), we can write the resulting Euclidean distance as:

$$\begin{aligned}
\Delta^2 &= \alpha^2 e^{-at}\left[1 - e^{-\delta t/2}\right]^2 + \left[e^{-at}\left\{\beta\left(1 - e^{-\delta t}\right) + \left(\frac{1}{a} - \frac{1}{a+\delta}e^{-\delta t}\right)\right\} - \left(\frac{1}{a} - \frac{1}{a+\delta}\right)\right]^2 \\
\Delta^2_{t \gg 1} &\approx e^{-at}\left[\alpha^2 - 2\left(\beta + \frac{1}{a}\right)\frac{\delta}{a(a+\delta)}\right] + \left[\frac{\delta}{a(a+\delta)}\right]^2
\end{aligned}$$

In the last line we have once again taken the $t \gg 1$ limit, where all terms decaying faster than e^{-at} are discarded. This last expression is maximised by setting $\alpha^2 = 1 - \beta^2$ in the first square bracket and finding its extremum:

$$\text{Maximise: } 1 - \beta^2 - 2\left(\beta + \frac{1}{a}\right)\frac{\delta}{a(a+\delta)} \implies \beta = -\frac{\delta}{a(a+\delta)}$$

Thus, we see that qubit states with *some* coherence is always optimal for finite temperatures, with the maximally coherent state (i.e. $\alpha = \pm 1$ and $\beta = 0$) being optimal for heatbaths with very similar temperatures (i.e. $\delta \to 0$). On the other hand, when the

hot reservoir has infinite temperature (i.e. $\delta \to \infty$), then states with coherence do not help, since in that case even the coherences damp away infinitely quickly. Finally, we can substitute this optimal β back into the expression for Δ^2 at late times to find:

$$\begin{aligned} \Delta^2_{t\gg 1,optimal} &= e^{-at}\left[1-\frac{\delta^2}{a^2(a+\delta)^2}-2\frac{a\delta}{a^2(a+\delta)^2}\right]+\left[\frac{\delta}{a(a+\delta)}\right]^2 \\ &= e^{-at}\left[1-\frac{\delta(\delta+2a)}{a^2(a+\delta)^2}\right]+\left[\frac{\delta}{a(a+\delta)}\right]^2 \end{aligned}$$

Note the expression in the first square bracket in the last equation is always strictly between $1/a^2$ and 1. So, Δ in this regime is always decreasing with time, indicating that the performance of the thermometer is better in this time regime, where the excited state population is damped but some coherence still persist, compared to full thermalisation.

Additional Data

It was noted in the text that whereas there were 9 and 10 datasets each for the $+Z$ and $+X$ input states, there were only 4 for $-Z$. Here we report an additional 5 that have been discarded due to hardware issues. Below, the equivalent of figure 3 is plotted for $-Z$, showing each dataset including censored ones. Evidently, data for the hot bath (triangular traces) appears to bifurcate. This occurred as a result of a hardware issue on a motorised waveplate mount during data taking, between runs.

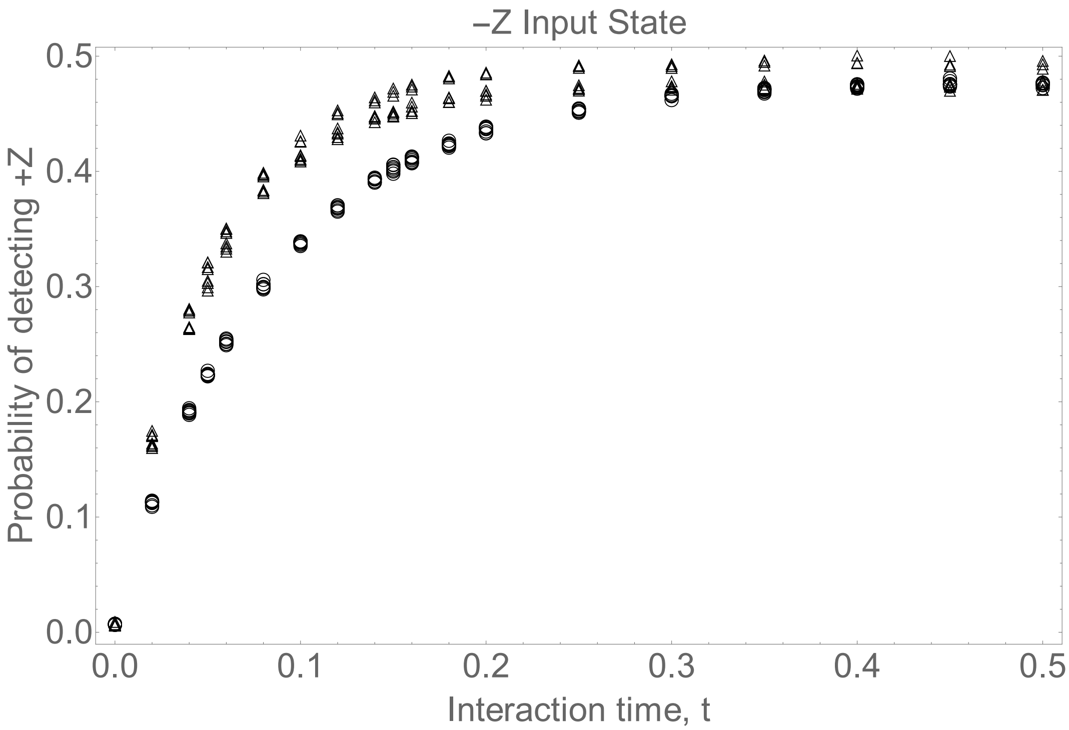

Figure 2. Plot of inferred probability of detecting $+Z$ given $-Z$ input state. Circular traces are for the cold bath whereas triangular traces are for the hot one.

Raw Adaptive Data

For the interested reader, we tabulate the probabilities of all 32 outcomes of a projector operator on 5 qubits under our adaptive scheme for discriminating $|1\rangle$ from $(|0\rangle+|1\rangle)/\sqrt{2}$. Table 1 shows theoretically computed probabilities whereas table 2 shows experimentally measured values. The first column in both table are indices that enumerate the projection outcome on the five qubits in the following sense: if we denote by 1 a successful projection and 0 a failed projection, and construct a binary string with the outcome of the first qubit on the left and the last on the right, then the little-endian integer representation of that binary string is the index shown in the first column of tables 1 and 2.

<table>
<tr><th></th><th>rho1</th><th>rho2</th><th>rho1</th><th>rho2</th><th>rho1</th><th>rho2</th><th>rho1</th><th>rho2</th><th>rho1</th><th>rho2</th></tr>
<tr><td>0</td><td rowspan="16">1.46E-01</td><td rowspan="16">8.54E-01</td><td rowspan="8">6.08E-02</td><td rowspan="8">8.47E-01</td><td rowspan="4">2.82E-02</td><td rowspan="4">8.46E-01</td><td rowspan="2">1.37E-02</td><td rowspan="2">8.46E-01</td><td>6.71E-03</td><td>8.46E-01</td></tr>
<tr><td>1</td><td>6.94E-03</td><td>5.50E-05</td></tr>
<tr><td>2</td><td rowspan="2">1.46E-02</td><td rowspan="2">2.35E-04</td><td>9.49E-07</td><td>1.20E-04</td></tr>
<tr><td>3</td><td>1.46E-02</td><td>1.16E-04</td></tr>
<tr><td>4</td><td rowspan="4">3.26E-02</td><td rowspan="4">1.09E-03</td><td rowspan="2">9.07E-06</td><td rowspan="2">5.62E-04</td><td>4.46E-06</td><td>5.62E-04</td></tr>
<tr><td>5</td><td>4.61E-06</td><td>3.66E-08</td></tr>
<tr><td>6</td><td rowspan="2">3.26E-02</td><td rowspan="2">5.26E-04</td><td>2.12E-06</td><td>2.67E-04</td></tr>
<tr><td>7</td><td>3.26E-02</td><td>2.59E-04</td></tr>
<tr><td>8</td><td rowspan="8">8.56E-02</td><td rowspan="8">6.15E-03</td><td rowspan="4">1.10E-04</td><td rowspan="4">3.29E-03</td><td rowspan="2">5.31E-05</td><td rowspan="2">3.29E-03</td><td>2.61E-05</td><td>3.29E-03</td></tr>
<tr><td>9</td><td>2.70E-05</td><td>2.14E-07</td></tr>
<tr><td>10</td><td rowspan="2">5.68E-05</td><td rowspan="2">9.16E-07</td><td>3.69E-09</td><td>4.65E-07</td></tr>
<tr><td>11</td><td>5.68E-05</td><td>4.51E-07</td></tr>
<tr><td>12</td><td rowspan="4">8.55E-02</td><td rowspan="4">2.85E-03</td><td rowspan="2">2.38E-05</td><td rowspan="2">1.47E-03</td><td>1.17E-05</td><td>1.47E-03</td></tr>
<tr><td>13</td><td>1.21E-05</td><td>9.59E-08</td></tr>
<tr><td>14</td><td rowspan="2">8.55E-02</td><td rowspan="2">1.38E-03</td><td>5.56E-06</td><td>7.01E-04</td></tr>
<tr><td>15</td><td>8.55E-02</td><td>6.78E-04</td></tr>
<tr><td>16</td><td rowspan="16">8.54E-01</td><td rowspan="16">1.46E-01</td><td rowspan="8">6.15E-03</td><td rowspan="8">8.56E-02</td><td rowspan="4">2.85E-03</td><td rowspan="4">8.55E-02</td><td rowspan="2">1.38E-03</td><td rowspan="2">8.55E-02</td><td>6.78E-04</td><td>8.55E-02</td></tr>
<tr><td>17</td><td>7.01E-04</td><td>5.56E-06</td></tr>
<tr><td>18</td><td rowspan="2">1.47E-03</td><td rowspan="2">2.38E-05</td><td>9.59E-08</td><td>1.21E-05</td></tr>
<tr><td>19</td><td>1.47E-03</td><td>1.17E-05</td></tr>
<tr><td>20</td><td rowspan="4">3.29E-03</td><td rowspan="4">1.10E-04</td><td rowspan="2">9.16E-07</td><td rowspan="2">5.68E-05</td><td>4.51E-07</td><td>5.68E-05</td></tr>
<tr><td>21</td><td>4.65E-07</td><td>3.69E-09</td></tr>
<tr><td>22</td><td rowspan="2">3.29E-03</td><td rowspan="2">5.31E-05</td><td>2.14E-07</td><td>2.70E-05</td></tr>
<tr><td>23</td><td>3.29E-03</td><td>2.61E-05</td></tr>
<tr><td>24</td><td rowspan="8">8.47E-01</td><td rowspan="8">6.08E-02</td><td rowspan="4">1.09E-03</td><td rowspan="4">3.26E-02</td><td rowspan="2">5.26E-04</td><td rowspan="2">3.26E-02</td><td>2.59E-04</td><td>3.26E-02</td></tr>
<tr><td>25</td><td>2.67E-04</td><td>2.12E-06</td></tr>
<tr><td>26</td><td rowspan="2">5.62E-04</td><td rowspan="2">9.07E-06</td><td>3.66E-08</td><td>4.61E-06</td></tr>
<tr><td>27</td><td>5.62E-04</td><td>4.46E-06</td></tr>
<tr><td>28</td><td rowspan="4">8.46E-01</td><td rowspan="4">2.82E-02</td><td rowspan="2">2.35E-04</td><td rowspan="2">1.46E-02</td><td>1.16E-04</td><td>1.46E-02</td></tr>
<tr><td>29</td><td>1.20E-04</td><td>9.49E-07</td></tr>
<tr><td>30</td><td rowspan="2">8.46E-01</td><td rowspan="2">1.37E-02</td><td>5.50E-05</td><td>6.94E-03</td></tr>
<tr><td>31</td><td>8.46E-01</td><td>6.71E-03</td></tr>
</table>

Table 1. Theoretically computed tabulation of outcome probabilities. Indices that enumerate rows begin at 0, and are integer representations of the outcomes of projective measurements on the qubits, in little-endian order (least significant bit is the last qubit).

	rho1	rho2	rho1	rho2	rho1	rho2	rho1	rho2	rho1	rho2
0	1.44E-01	8.52E-01	5.88E-02	8.44E-01	2.68E-02	8.40E-01	1.27E-02	8.36E-01	6.17E-03	8.32E-01
1									6.52E-03	4.08E-03
2							1.41E-02	3.59E-03	1.52E-05	1.79E-03
3									1.41E-02	1.80E-03
4					3.19E-02	3.96E-03	5.50E-05	2.02E-03	2.66E-05	2.01E-03
5									2.84E-05	8.00E-06
6							3.19E-02	1.95E-03	3.51E-05	9.77E-04
7									3.18E-02	9.72E-04
8			8.49E-02	7.91E-03	2.73E-04	4.18E-03	1.28E-04	4.16E-03	6.19E-05	4.15E-03
9									6.58E-05	1.71E-05
10							1.45E-04	1.67E-05	1.74E-07	8.33E-06
11									1.45E-04	8.40E-06
12					8.46E-02	3.73E-03	1.35E-04	1.90E-03	6.51E-05	1.89E-03
13									6.95E-05	8.39E-06
14							8.44E-02	1.83E-03	8.67E-05	9.18E-04
15									8.44E-02	9.15E-04
16	8.56E-01	1.48E-01	8.61E-03	8.52E-02	3.92E-03	8.49E-02	1.87E-03	8.45E-02	9.08E-04	8.41E-02
17									9.57E-04	3.81E-04
18							2.05E-03	3.61E-04	2.48E-06	1.81E-04
19									2.05E-03	1.80E-04
20					4.69E-03	3.59E-04	6.65E-06	1.82E-04	3.20E-06	1.82E-04
21									3.45E-06	7.73E-07
22							4.69E-03	1.77E-04	6.06E-06	8.82E-05
23									4.68E-03	8.89E-05
24			8.48E-01	6.28E-02	2.59E-03	3.31E-02	1.22E-03	3.30E-02	5.90E-04	3.28E-02
25									6.30E-04	1.39E-04
26							1.37E-03	1.37E-04	1.52E-06	6.87E-05
27									1.36E-03	6.86E-05
28					8.45E-01	2.97E-02	1.57E-03	1.51E-02	7.55E-04	1.50E-02
29									8.13E-04	6.74E-05
30							8.44E-01	1.46E-02	9.26E-04	7.32E-03
31									8.43E-01	7.30E-03

Table 2. Tabulation of experimental outcome probabilities. Indices that enumerate rows begin at 0, and are integer representations of the outcomes of projective measurements on the qubits, in little-endian order (least significant bit is the last qubit).
